# Comparative analgesic effect of Ligusticum chuanxiong pieces and its products in mice

**DOI:** 10.4103/0973-1296.62902

**Published:** 2010-05-05

**Authors:** Demin GAO, Lingchuan XU

**Affiliations:** *School of Pharmacy, Shandong University of Traditional Chinese Medicine, 250355 Jinan, China*

**Keywords:** Analgesic effect, chuanxiong, dysmenorrhea test, hot plate test, writhing test

## Abstract

The present study was undertaken with the objective of finding out the comparative analgesic effect of Ligusticum chuanxiong (LC) pieces decoction, LC formula granule decoction, liquored LC pieces decoction and liquored LC formula granule decoction. The analgesic effects were analyzed using the hot plate and acetic-induced writhing test in mice, and antidysmenorrheic effect was observed with primary dysmenorrhea model. The results showed that four kinds of LC decoction had definite effect in delaying incubation period and decreasing the writhing frequency within 30 min. They also effectively relieved dysmenorrhea. Moreover, liquored LC had better analgesic effect than crude LC in four decoctions.

## INTRODUCTION

The rhizome of Ligusticum chuanxiong Hort. (LC), a well-known traditional Chinese medicinal herb, is widely used for the treatment of ailments such as irregular menses, dysmenorrhea, pectoralgia, rheumatic arthralgia, etc.[1] In clinic, it has different medication forms, such as crude LC pieces, LC formula granule, liquored LC pieces and liquored LC formula granule, etc. However, their pharmacological activities are not yet fully understood. In this article, to explore the analgesic effect of rhizoma of LC, four LC agents were investigated using the hot plate and mouse writhing test. Subsequently, their analgesic effect was further assessed with dysmenorrhea model.

## MATERIALS AND METHODS

### Reagents and animals

LC pieces, LC formula granule and their liquored products were obtained from Green Pharmaceutical Technology Corporation (Sichuan, China); Aspirin was acquired from Dayang Pharmaceutical Technology Corporation (Beijing, China); Indometacin and oxytocin were purchased from Jinquan Biotechnology Corporation (Dalian, China) and Biochemistry Pharmaceutical Corporation (Tianjin, China) respectively. Kunming mice were obtained from Experimental Animal Center of Shandong University.

### Decoction preparation

LC pieces decoction and its liquored pieces decoction were prepared based on the general preparation in the Chinese Pharmacopoeia. Viz. LC pieces were macereted in water for 30 min, and subsequently, the mixture was repeatedly boiled and filtered, and the combined filtrate was concentrated to 0.2 g·ml^−1^ crude drug. LC formula granule and its liquored formula granule were heated to solution and concentrated to 0.2 g·ml^−1^ crude drug.

### Hot plate test

The hot plate test was used to measure reaction time, as described by Eddy and Leimback.[2] In the experiment, the hot plate (Beijing GENE and life science Corporation) was maintained at 55 ± 0.1 °C. Sixty mice were randomly divided into negative control group, positive control group, LC pieces group, LC formula granule group, liquored LC pieces group and liquored LC formula granule group, with 10 mice in each group. Kunming mouse were preselected, and animals showing a reaction time greater than 30 s or less than 5 s were discarded. The reaction time was determined by observing either the licking of the hind paws or the jumping movements before and 60 m, 120 m and 180 m after intragastric administration of the four LC agents (0.015 ml g^−1^ body). Normal saline and aspirin (0.015 ml.g^−1^ body), were used as the negative and positive controls, respectively.

### Writhing test

The writhing test is described in detail elsewhere.[3,4] Half an hour after the intragastric administration of the medicine (0.015 ml g^−1^ body) or normal saline, each mouse was given an intraperitoneal injection of 0.2 ml acetic acid solution (0.7%, v/v). The mice were placed individually into glass beakers and five minutes later, the writhing response consisted of a contraction of the abdominal muscles together with a stretching of the hind limbs. The latency and the number of writhing movements made by each mouse were counted for 30 min.

### Primary dysmenorrhea model test

Sixty female mice were randomly assigned into six groups. Each mouse was administrated intragastrically 0.2 mg diethylstilbestrol once daily for 12 days. On the sixth day, medicinal agents were administrated (i.g.) 0.015 ml·g^−1^ body once daily for 6 days. Ocytocin (i.p. 200 U.g^−1^) was injected on the twelfth day 30 min later after the last administration.[5] The controls used were normal saline (0.015 ml·g^−1^) and indometacin (0.015 ml·g^−1^) respectively. The latency and incidence rate of writhing within 30 min were observed.

## RESULTS

### Effect of LC decoction on the hot plate response

**Figure 1:**
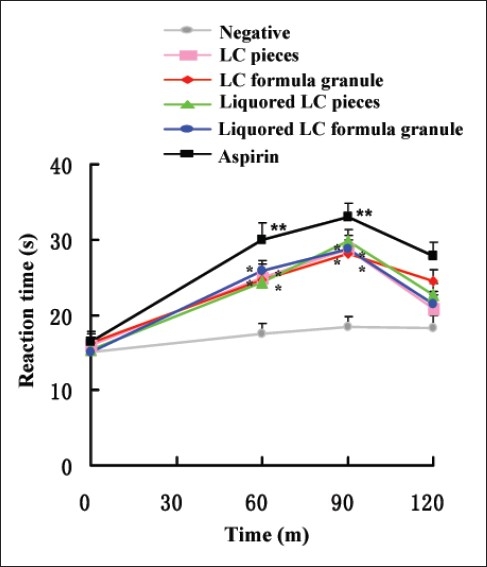
Effect of LC decoction on the hot plate response in mice. Mice were pretreated with normal saline (negative control), aspirin (positive control), LC pieces decoction, LC formula granule decoction, liquored LC pieces decoction and liquored LC formula granule decoction (each agent, 0.015 mL g-1), Reaction time were measured before intragastric administration of the above agents and 60, 90 and 120 m afterward. Data were reported as means±S.E.M for the groups of 10 mice. *P<0.05 and **P<0.01 compared to the negative control as determined by the Studentæs t-test

The results [Figure 1] showed that LC decoction significantly increased reaction time for nociception above the control value from the beginning to 90 min (*P<0.05, **P<0.01). Among the LC decoction groups, liquored LC decoction had better analgesic effect than crude LC decoction, but no significant difference appeared in the experiment.

### Effect of LC decoction on the writhing response

**Figure 2:**
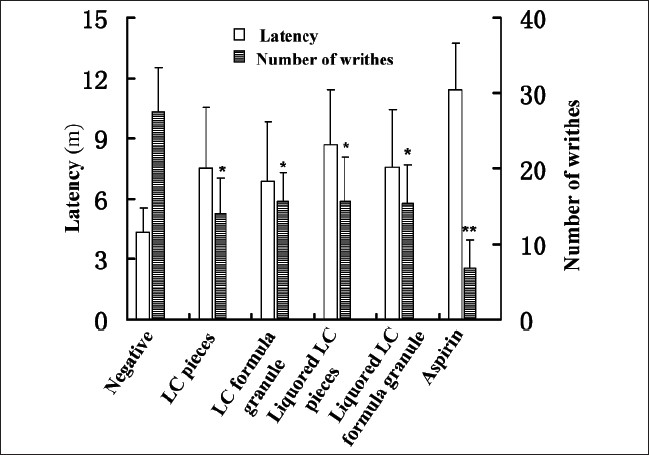
Effect of LC decoction on the acetic acid-induced writhing response. LC decoction (0.015 mL g-1 body) were given by intragastric administration in mice. 30 m after the administration of medicine, animals received an i.p. injection of 0.2 mL acetic acid solution (0.7%, v/v). The latency and the number of writhes in the following 30 m was determined. Control experiments were performed using normal saline and aspirin. Results were expressed as means±S.E.M for the groups of 10 mice. *P<0.05 and **P<0.01 compared to the negative control as determined by the Studentæs t-test

The Figure 2 indicated the longer latency and the less number of writhes in the medicinal groups, compared with the negative control group, and the aspirin was the best analgesic drug among the six groups. The experiment also showed that the liquored pieces decoction had better analgesic effect than crude LC pieces decoction.

### Effect of LC decoction on mouse primary dysmenorrhea model

**Figure 3:**
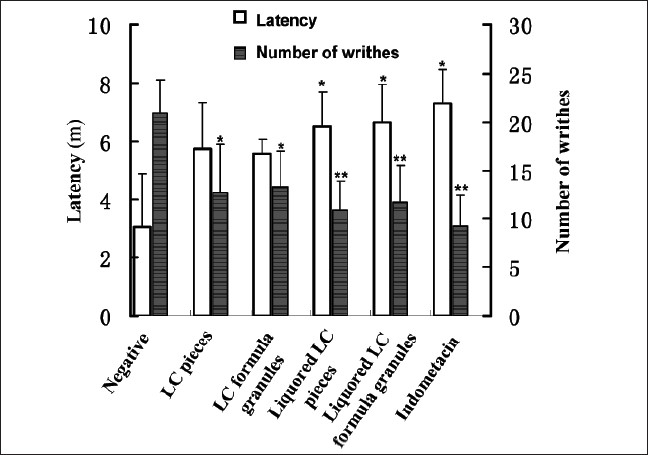
Effect of LC decoction on mouse primary dysmenorrhea model. Primary dysmenorrheal model was established by intragastric administration of 0.2 mg diethylstilbestrol once daily for 12 days. Medicinal agent was given to each mouse, once daily for 6 days from the 6th day on after modeling. On the 12th day, ocytocin (i.p. 0.2 U/kg) was injected to mouse. The latency and incidence rate of writhing within 30 min were observed. Results were expressed as means±S.E.M for the groups of 10 mice. *P<0.05 and **P<0.01 compared to the negative control as determined by the Studentæs t-test

Compared with the negative control, LC decoction can effectively relieve dysmenorrhea and prolong the incubation period caused by oxytocin. Moreover, liquored LC decoction had a greater advantage of analgesic effect than crude LC decoction [Figure 3].

## DISCUSSION

It is well known that the LC could extend blood vessel, improve cerebral circulation, suppress platelet aggregation and relieve pain. The experiments showed that crude LC pieces and its processed products had similar analgesic effects, but no statistic significance appeared between the four medicinal agents described above, which partly explained the fact that there existed both LC rhizoma pieces and processed LC products in clinical medicine. The results also documented that liquored LC products had better analgesic effect than crude LC pieces, which may be due to their slightly different constituents.[6–8] Because of the fried process with the yellow wine, parts of constituents of crude LC might have changed. To further explore the mechanism of action of LC formulas and decoctions, other pharmacodynamics including analgesic effects of remaining crude LC and its preparations need to be researched.

In the present study, the dose of LC decoction for the animal was the largest dosage, which was derived from the experimental study[9] and clinical application.[10] Based on which, as well as its atoxic characters, medium dosage and low dosage experiments on animals were not carried out.

In summary, our study demonstrated the fact that liquored LC pieces or liquored LC formula granule had better analgesic effect than crude LC pieces in clinic.
